# Hyperkalemic effect of drug–drug interaction between esaxerenone and trimethoprim in patients with hypertension: a pilot study

**DOI:** 10.1186/s40780-024-00366-6

**Published:** 2024-08-02

**Authors:** Toshinori Hirai, Shun Ueda, Toru Ogura, Kan Katayama, Kaoru Dohi, Yuki Kondo, Yuka Sakazaki, Yoichi Ishitsuka, Takuya Iwamoto

**Affiliations:** 1 Department of Pharmacy, Mie University Hospital, Faculty of Medicine, Mie University, 2-174 Edobashi, Tsu, Mie 514-8507 Japan; 2 Clinical Research Support Center, Mie University Hospital, 2-174 Edobashi, Tsu, Mie 514-8507 Japan; 3 Department of Cardiology and Nephrology, Mie University Graduate School of Medicine, Mie University Hospital, 2-174 Edobashi, Tsu, Mie 514-8507 Japan; 4 Department of Clinical Chemistry and Informatics, Graduate School of Pharmaceutical Sciences, Kumamoto University, 5-1 Oehonmachi, Chuo-ku, Kumamoto, Kumamoto 862-0973 Japan

**Keywords:** Drug–drug interaction, Esaxerenone, Trimethoprim, Potassium, Hyperkalemia

## Abstract

**Background:**

We examined whether the pharmacodynamic drug–drug interaction between esaxerenone and trimethoprim enhances the hyperkalemic effect.

**Methods:**

A retrospective observational study was conducted to identify patients >18 years undertaking esaxerenone alone or esaxerenone plus trimethoprim at Mie University Hospital from May 2019 to December 2022. We performed propensity score-matching (1:1) to compare between-group differences in the maximum change in serum potassium levels (ΔK) using the Mann–Whitney U test. For esaxerenone plus trimethoprim, Spearman's correlation coefficients were used to examine correlations between ΔK and variables, including changes in blood urea nitrogen (ΔBUN), serum creatinine levels (ΔCr), and weekly trimethoprim cumulative dose.

**Results:**

Out of propensity score-matched groups (*n*=8 each), serum potassium levels significantly increased after administration of esaxerenone alone (4.4 [4.2 to 4.7] meq/L to 5.2 [4.7 to 5.4] meq/L, *p*=0.008) and esaxerenone plus trimethoprim (4.2 [4.0 to 5.1] meq/L to 5.4 [4.7 to 5.5] meq/L, *p*=0.023). ΔK did not significantly differ between the groups (esaxerenone alone; 0.6 [0.3 to 0.9] meq/L vs. esaxerenone plus trimethoprim; 1.0 [0.4 to 1.3] meq/L, *p*=0.342). ΔK positively correlated with ΔBUN (r=0.988, *p*<0.001) or ΔCr (r=0.800, *p*=0.017). There was a trend of correlation of ΔK with a weekly cumulative trimethoprim dose (r=0.607, *p*=0.110).

**Conclusions:**

The hyperkalemic effect of the drug–drug interaction between esaxerenone and trimethoprim is not notable and related to renal function and trimethoprim dosage.

**Supplementary Information:**

The online version contains supplementary material available at 10.1186/s40780-024-00366-6.

## Background

Esaxerenone (a mineralocorticoid receptor antagonist) blocks aldosterone binding to mineralocorticoid receptors in the distal convoluted tubule and collecting duct, thereby inhibiting sodium reabsorption and potassium secretion [1]. Randomized controlled trials have shown that esaxerenone lowers blood pressure and urinary protein level in patients with essential hypertension, type 2 diabetes, and microalbuminuria [2]. However, hyperkalemia occurs during esaxerenone treatment in approximately up to 10% of patients [2, 3], causing lethal arrhythmias and cardiac arrest.

Aldosterone-activated epithelial sodium channels are key regulators of potassium excretion [4]. Since animal studies demonstrated that trimethoprim inhibits epithelial sodium channels in a concentration-dependent manner [5], trimethoprim can amplify the hyperkalemic effect of esaxerenone in humans. Previous studies have suggested a higher risk of sudden death and hyperkalemia with the co-administration of spironolactone (a traditional mineralocorticoid receptor antagonist) and trimethoprim [6, 7]. In particular, esaxerenone has a high inhibitory capacity for mineralocorticoid receptors and a long half-life among mineralocorticoid receptor antagonists (3.7 nM and 18.6 h, respectively) [8]. We have identified that the hyperkalemic effect is obvious when esaxerenone and clarithromycin are co-administered, but there was no increase in serum potassium level in patients with eplerenone and clarithromycin, owing to the inhibition of cytochrome P450 3A4 metabolism for mineralocorticoid receptor antagonist by clarithromycin [9]. Therefore, we hypothesized that the hyperkalemic effect would be greater in patients who received esaxerenone and trimethoprim concomitantly than in those who received esaxerenone alone.

The objective of this study was to evaluate hyperkalemia due to the pharmacodynamic interaction between esaxerenone and trimethoprim.

## Methods

### Study design

This single-center retrospective observational study enrolled all patients (>18 years) who received esaxerenone alone or esaxerenone plus trimethoprim at Mie University Hospital from May 2019 to December 2022. Exclusion criteria included non-measurement of serum potassium levels or serum potassium levels exceeding 5.5 meq/L at baseline, according to the Common Terminology Criteria for Adverse Events version 5.0. The Institutional Review Board of Mie University Hospital approved the study protocol in accordance with the Declaration of Helsinki (H2023-075).

### Data collection

Data collection through electronic medical chart review included demographics, co-existing diseases, clinical laboratory data, test drugs (esaxerenone and trimethoprim), and concomitant medications known to alter serum potassium levels (angiotensin receptor neprilysin inhibitor, renin-angiotensin system inhibitors, loop diuretics, thiazide diuretics, and sodium-glucose co-transporter 2 inhibitors). The follow-up period was till the point of treatment termination with esaxerenone alone and esaxerenone plus trimethoprim, or December 2022. The blood samples were collected in the morning. The estimated glomerular filtration rate (eGFR) was calculated as follows [10]:

$$\text{eGFR }\left(\text{mL}/\text{min}/{1.73\text{m}}^{2}\right)=194\times {\text{age}}^{-0.287}\times {\text{serum creatinine}}^{-1.094}\times 0.739 \left(\text{if female}\right)$$

### Outcomes

The primary outcome was the maximum change in serum potassium levels (ΔK, meq/L) from baseline to the maximum value at post-dose as calculated by the following equation:

$$\Delta \text{K }\left(\text{meq}/\text{L}\right)=\text{K at maximum value}-\text{K at baseline}$$

When pseudo-hyperkalemia (e.g., blood collection technique or hemolysis) was present, we alternatively used remeasured serum potassium levels for the analysis. Likewise, the secondary outcome comprised changes in serum sodium levels (ΔNa, meq/L), blood urea nitrogen (ΔBUN, mg/dL), serum creatinine levels (ΔCr, mg/dL), and eGFR (ΔeGFR, mL/min/1.73m^2^) at the point of maximum potassium levels since trimethoprim potentially causes hyponatremia and pseudo-elevation of serum creatinine [11, 12].

### Statistical analysis

Statistical analysis was conducted using JMP^®^ Pro 16.2.0 (SAS Institute Inc., Cary, NC, USA). Statistical significance was a two-tailed *p*-value <0.05. Data were presented as median [interquartile range (IQR)] for continuous data or as numbers (%) for categorical data. Continuous data were compared using the Mann–Whitney U test (unpaired data) or Wilcoxon signed-rank test (paired data). The heterogeneity of categorical data was evaluated using the chi-square test. Spearman's correlation coefficients were used to analyze the correlations between continuous variables.

A 1:1 propensity score-matched pair was generated to adjust for potential confounders based on the propensity score as a probability of receiving esaxerenone plus trimethoprim in logistic regression analysis. The variables in the propensity score included sex, age, body mass index, serum potassium, serum sodium, serum chloride, albumin, blood urea nitrogen, eGFR, esaxerenone dosing schedule, angiotensin receptor neprilysin inhibitor or renin-angiotensin system inhibitors, loop and thiazide diuretics, and sodium-glucose co-transporter 2 inhibitors.

We compared the outcomes of within-group differences in each group and between-group differences between esaxerenone alone and esaxerenone plus trimethoprim. A sub-analysis was conducted to examine the correlations between ΔK and variables such as secondary outcomes and weekly cumulative trimethoprim dose in the esaxerenone plus trimethoprim group. Since the kidney plays an essential role in eliminating trimethoprim [13], the weekly cumulative trimethoprim dose normalized by eGFR was also used for sub-analysis.

## Results

### Clinical characteristics

Of the 185 patients who received esaxerenone (esaxerenone alone, *n*=177; esaxerenone plus trimethoprim, *n*=8), 72 met the exclusion criteria. Ultimately, 113 patients were included in the analysis (esaxerenone alone, *n*=105; esaxerenone plus trimethoprim, *n*=8). Serum albumin, blood urea nitrogen, and esaxerenone dosing schedules differed between the groups significantly ﻿(Additional data 1). After propensity score-matching, the clinical characteristics were well-balanced (*n*=8 each; Table 1). The dosing schedule of esaxerenone comprised a low dose (0.625 mg×1, 1.25 mg×1, and 2.5 mg ×1) in both groups. In the esaxerenone plus trimethoprim group, all patients prophylactically received cotrimoxazole containing 400 mg of sulfamethoxazole and 80 mg of trimethoprim in one tablet. The trimethoprim dosing schedule was daily (one tablet ×2 every day; *n*=1) and non-daily (half tablet ×1 twice a week; *n*=1 and one tablet ×1 thrice a week; *n*=6). The median follow-up duration was 186 [IQR: 47 to 292] days for esaxerenone alone and 95 [23 to 222] days for esaxerenone plus trimethoprim (*p*=0.25).

**Table 1:** Clinical characteristics after propensity score-matching

**Variables**	**ESA alone, *n*=8**	**ESA+TMP, *n*=8**	***P* value**
**Demographical data**			
Male, n (%)	5 (63)	3 (38)	0.32
Age, years	78 [70 to 86]	72 [64 to 75]	0.11
Body weight, kg	64 [55 to 76]	56 [51 to 78]	0.24
Body mass index, kg/m^2^	23 [19 to 27]	23 [20 to 26]	0.75
**Co-existing disease**			
Hypertension, n (%)	8 (100)	8 (100)	-
Heart failure, n (%)	4 (50)	2 (25)	0.30
Diabetes, n (%)	1 (13)	3 (38)	0.25
**Clinical laboratory data**			
Serum potassium, meq/L	4.4 [4.2 to 4.7]	4.2 [4.0 to 5.1]	0.29
Serum sodium, meq/L	140 [139 to 143]	141 [139 to 143]	0.46
Serum chloride, meq/L	106 [104 to 108]	109 [104 to 110]	0.43
Serum albumin, g/dL	3.7 [3.0 to 3.9]	3.4 [2.9 to 3.7]	0.56
Blood urea nitrogen, mg/dL	25.1 [21.0 to 30.5]	21.2 [17.4 to 26.6]	0.25
Serum creatinine, mg/dL	1.29 [1.00 to 1.82]	1.00 [0.74 to 1.31]	0.12
eGFR, mL/min/1.73m^2^	38.9 [27.8 to 45.4]	50.9 [38.8 to 63.3]	0.09
**ESA**			
0.625 mg ×1, n (%)	0 (0)	1 (13)	0.30
1.25 mg ×1, n (%)	7 (88)	5 (63)	0.25
2.5 mg ×1, n (%)	1 (13)	2 (25)	0.52
3.75 mg ×1, n (%)	0 (0)	0 (0)	-
5.0 mg ×1, n (%)	0 (0)	0 (0)	-
**TMP**			-
Daily administration, n (%)	-	1 (13)	
Non-daily administration, n (%)	-	7 (88)	
**Medications**			
ARNI or RASi, n (%)	8 (100)	6 (75)	0.13
Loop diuretics, n (%)	2 (25)	2 (25)	1.00
Thiazide diuretics, n (%)	1 (13)	1 (13)	1.00
SGLT2i, n (%)	0 (0)	0 (0)	-

*Abbreviations: ESA* esaxerenone, *TMP* trimethoprim, *eGFR* estimated glomerular filtration rate, *ARNI* angiotensin receptor neprilysin inhibitor, *RASi* renin-angiotensin system inhibitor, *SGLT2i* sodium-glucose co-transporter 2 inhibitor. Continuous data are presented as median [interquartile range] and compared using the Mann–Whitney U test. Categorical data are presented as numbers (%) and evaluated using the chi-square test

$$\text{eGFR }\left(\text{mL}/\text{min}/{1.73\text{m}}^{2}\right)=194\times {\text{age}}^{-0.287}\times {\text{serum creatinine}}^{-1.094}\times 0.739 \left(\text{if female}\right)$$ [10]

### Outcome

Table 2 summarizes the study outcomes. Serum potassium levels significantly increased after esaxerenone alone from 4.4 [4.2 to 4.7] meq/L to 5.2 [4.7 to 5.4] meq/L (*p*=0.008) and esaxerenone plus trimethoprim from 4.2 [4.0 to 5.1] meq/L to 5.4 [4.7 to 5.5] meq/L (*p*=0.023). No significant difference in ΔK was found between the esaxerenone alone and esaxerenone plus trimethoprim (esaxerenone alone: 0.6 [0.3 to 0.9] meq/L vs. esaxerenone plus trimethoprim: 1.0 [0.4 to 1.3] meq/L, *p*=0.342).

**Table 2:** Summary of outcomes

	**n**	**Before**	**After**	**Δ**	***P* value (Before vs after)**	***P* value (Between groups)**
**K, meq/L**						
ESA alone	8	4.4 [4.2 to 4.7]	5.2 [4.7 to 5.4]	0.6 [0.3 to 0.9]	0.008	0.342
ESA+TMP	8	4.2 [4.0 to 5.1]	5.4 [4.7 to 5.5]	1.0 [0.4 to 1.3]	0.023	
**Na, meq/L**						
ESA alone	8	140 [139 to 143]	141 [139 to 142]	-1 [-2 to 1]	0.375	0.749
ESA+TMP	8	141 [139 to 143]	141 [138 to 143]	-1 [-3 to 1]	0.266	
**BUN, mg/dL**						
ESA alone	8	25.1 [21.0 to 30.5]	30.5 [23.0 to 33.9]	5.7 [-0.7 to 7.0]	0.055	0.674
ESA+TMP	8	21.2 [17.4 to 26.6]	28.5 [19.0 to 34.3]	4.1 [-0.5 to 11.0]	0.109	
**Cr, mg/dL**						
ESA alone	8	1.29 [1.00 to 1.82]	1.65 [1.03 to 2.17]	0.23 [0.02 to 0.45]	0.039	0.752
ESA+TMP	8	1.00 [0.74 to 1.31]	1.31 [0.76 to 1.60]	0.20 [-0.03 to 0.43]	0.086	
**eGFR, mL/min/1.73m**^**2**^						
ESA alone	8	38.9 [27.8 to 45.4]	31.2 [23.4 to 41.0]	-3.4 [-8.3 to -0.9]	0.039	0.401
ESA+TMP	8	50.9 [38.8 to 63.3]	40.1 [30.0 to 60.9]	-9.8 [-14.7 to 1.4]	0.055	

*Abbreviations: K* potassium, *Na* sodium, *BUN* blood urea nitrogen, *Cr* creatinine, *eGFR* estimated glomerular filtration rate, *ESA* esaxerenone, *TMP* trimethoprim

Data are presented as the median [interquartile range]. Data are compared using the Mann–Whitney U test (between groups) or Wilcoxon signed-rank test (before vs. after)

$$\Delta =\text{maximum value}-\text{baseline value}$$

The maximum value is at the observation of the maximum potassium value

$$\text{eGFR }\left(\text{mL}/\text{min}/{1.73\text{m}}^{2}\right)=194\times {\text{age}}^{-0.287}\times {\text{serum creatinine}}^{-1.094}\times 0.739 \left(\text{if female}\right)$$ [10]

Similarly, there were no significant difference in ΔNa (esaxerenone alone: -1 [-2 to 1] meq/L vs. esaxerenone plus trimethoprim: -1 [-3 to 1] meq/L, *p*=0.749), ΔBUN (esaxerenone alone: 5.7 [-0.7 to 7.0] mg/dL vs. esaxerenone plus trimethoprim: 4.1 [-0.5 to 11.0] mg/dL, *p*=0.674), ΔCr (esaxerenone alone: 0.23 [0.02 to 0.45] mg/dL vs. esaxerenone plus trimethoprim: 0.20 [-0.03 to 0.43] mg/dL, *p*=0.752), and ΔeGFR (esaxerenone alone: -3.4 [-8.3 to -0.9] mL/min/1.73m^2^ vs. esaxerenone plus trimethoprim: -9.8 [-14.7 to 1.4] mL/min/1.73m^2^, *p*=0.401). The serum sodium levels were comparable after esaxerenone alone from 140 [139 to 143] meq/L to 141 [139 to 142] meq/L (*p*=0.375) and esaxerenone plus trimethoprim from 141 [139 to 143] meq/L to 141 [138 to 143] meq/L (*p*=0.266). Blood urea nitrogen did not differ after esaxerenone alone from 25.1 [21.0 to 30.5] mg/dL to 30.5 [23.0 to 33.9] mg/dL (*p*=0.055) and esaxerenone plus trimethoprim from 21.2 [17.4 to 26.6] mg/dL to 28.5 [19.0 to 34.3] mg/dL (*p*=0.109). Serum creatinine levels significantly increased after esaxerenone alone from 1.29 [1.00 to 1.82] mg/dL to 1.65 [1.03 to 2.17] mg/dL (*p*=0.039), although there was no significant increase after esaxerenone plus trimethoprim from 1.00 [0.74 to 1.31] mg/dL to 1.31 [0.76 to 1.60] mg/dL (*p*=0.086). A significant decrease in eGFR was also observed in esaxerenone alone from 38.9 [27.8 to 45.4] mL/min/1.73m^2^ to 31.2 [23.4 to 41.0] mL/min/1.73m^2^ (*p*=0.039), whereas an insignificant decrease was detected after esaxerenone plus trimethoprim from 50.9 [38.8 to 63.3] mL/min/1.73m^2^ to 40.1 [30.0 to 60.9] mL/min/1.73m^2^ (*p*=0.055).

**Fig. 1:**
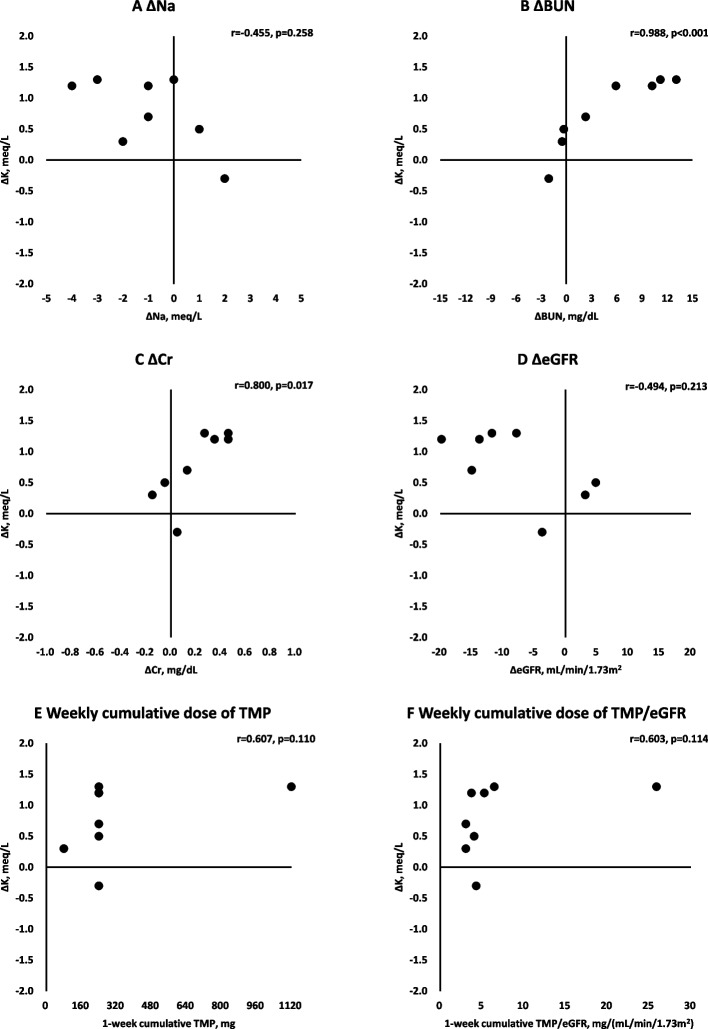
Factors influencing maximum serum potassium level changes in patients receiving esaxerenone and trimethoprim Abbreviations: K, potassium; Na, sodium; BUN, blood urea nitrogen; Cr, creatinine; eGFR, estimated glomerular filtration rate; TMP, trimethoprim. The y- and x-axes represent maximum changes in serum potassium levels (ΔK) and changes in Na, BUN, Cr, and eGFR at the timing of maximum serum potassium level, and TMP dosing (weekly cumulative TMP and weekly cumulative TMP/eGFR), respectively. $\triangle K(meq/L)=K\;at\;maximum\;value-K\;at\;baseline$ The maximum value was at the observation of the maximum potassium value. $\text{eGFR }\left(\text{mL}/\text{min}/{1.73\text{m}}^{2}\right)=194\times {\text{age}}^{-0.287}\times {\text{serum creatinine}}^{-1.094}\times 0.739 \left(\text{if female}\right)$ [10]. **A** ΔNa, **B** ΔBUN, **C** ΔCr, **D** ΔeGFR, **E** Weekly cumulative dose of TMP, **F** Weekly cumulative dose of TMP/eGFR

We confirmed significant correlations of ΔK with ΔBUN (Fig. 1B, r =0.988, *p*<0.001) and ΔCr (Fig. 1C, r=0.800, *p*=0.017). Additionally, ΔK tended to correlate with the weekly cumulative trimethoprim dose (Fig. 1E, r=0.607, *p* =0.110) and the weekly cumulative trimethoprim dose normalized by eGFR (Fig. 1F, r=0.603, *p* =0.114).

## Discussion

The hyperkalemic effects of esaxerenone alone and esaxerenone plus trimethoprim were comparable in a small size of the study. Moreover, ΔK significantly correlated with ΔBUN and ΔCr. In addition, the trimethoprim dose may have enhanced the hyperkalemic effect after treatment with esaxerenone and trimethoprim. These insights will help to continue the treatment of esaxerenone and avoid the risk of critical hyperkalemia due to the pharmacodynamic interaction between esaxerenone and trimethoprim.

A randomized controlled trial reported that the median increase in serum potassium levels was 0.2 meq/L [2]. In contrast, the hyperkalemic effect was notable in patients taking low-dose esaxerenone with and without trimethoprim (Table 2). We postulated that older age and reduced eGFR exacerbated the hyperkalemic effect. Compared with other landmark trials, the patients in our study were older (≥70 years vs. <70 years) [2]. Advancing age is a risk factor for hyperkalemia associated with spironolactone, a mineralocorticoid receptor antagonist [14]. Similarly, our study patients had lower eGFR (40 to 50 mL/min/1.73m^2^ vs. approximately 70 mL/min/1.73m^2^) [2]. In particular, a clinical trial set the exclusion criteria for eGFR <60 mL/min/1.73m^2^ [2]. Since the urinary excretion of potassium is determined by kidney function [15], clinicians should evaluate renal function following esaxerenone treatment.

Our finding contradicted the hypothesis. This reason is that the co-administration of trimethoprim at a prophylactic dose did not influence the hyperkalemic effects of esaxerenone. We speculated that the potential mechanism of drug–drug interaction involves aldosterone antagonism by esaxerenone [1] and epithelial sodium channel blockade by trimethoprim [5]. Some case reports have shown severe hyperkalemia in patients receiving trimethoprim under hypoaldosteronism [16, 17]. This phenomenon is similar to the effects of drug–drug interactions between esaxerenone and trimethoprim. In fact, esaxerenone at 1.25 to 5.0 mg ×1 secondary increased plasma renin activity and plasma aldosterone concentration in essential hypertension or primary aldosteronism [18, 19], which is reflective of hypoaldosteronism. Our study showed that the absolute ΔK was numerically larger in patients administered esaxerenone plus trimethoprim than in those administered esaxerenone alone, indicating that the pharmacodynamic interaction between esaxerenone and trimethoprim is clinically significant to a greater or lesser extent. From the viewpoint of potassium kinetics, total potassium has a positive correlation with body size that stores potassium in the body [20]. Indeed, we previously reported that a higher hyperkalemic risk of renin-angiotensin system inhibitors was observed in patients with reduced renal function in addition to low body size [21]. In this study, the group of esaxerenone and trimethoprim had -9.8 mL/min/1.73m^2^ of ΔeGFR from baseline (Table 2). The absolute ΔK might depend on the kidney function.

Trends toward reduced eGFR were observed in both the esaxerenone alone and esaxerenone plus trimethoprim groups. Trimethoprim inhibits creatinine secretion via organic cation transporter 2, which increases serum creatinine levels at an average of 0.05 to 0.2 mg/dL or 6 to 20% from baseline [22–24]. In the present study, ΔCr was within the reported range. The pseudo-elevation of serum creatinine by trimethoprim did not directly affect renal function [25–27]. The magnitude of ΔK was higher, especially when ΔCr exceeded an average elevation of serum creatinine level in pseudo-elevation (0.2 mg/dL) (Fig. 1C). Since the kidney handles the elimination of potassium and trimethoprim [13, 15], serum creatinine level is a valuable marker for estimating the magnitude of drug–drug interactions between esaxerenone and trimethoprim in case of ΔCr ≥0.2 mg/dL. Furthermore, ΔK significantly correlated with ΔBUN as well as ΔCr. Blood urea nitrogen homeostasis depends on diet (protein), catabolism, and glomerular filtration [28]. Unlike creatinine [29], urea reabsorption increases with decreasing urine flow rate mediated by vasopressin in the collecting duct [30]. Therefore, ΔBUN may indirectly reflect slow urine flow and low capacity for potassium excretion.

Sub-analysis revealed a mild but non-significant correlation between ΔK and weekly cumulative trimethoprim dose (Fig. 1E and F). Trimethoprim impairs potassium excretion in the urine by inhibiting epithelial sodium channels in a concentration-dependent manner [5]. Although a therapeutic dose of trimethoprim (>10 mg/kg/day) is likely to cause hyperkalemia powerfully [31], prophylactic doses (e.g., one tablet ×1) could also cause hyperkalemia [32, 33]. Almost all patients in the present analysis received non-daily administration of trimethoprim as prophylaxis. Recently, a low dose of cotrimoxazole at a daily dose of 100 mg sulfamethoxazole and 20 mg trimethoprim was found to be an acceptable option for Pneumocystis pneumonia prophylaxis after transplantation [34]. Therefore, it is required to confirm whether co-administration of a minimal dose of trimethoprim (i.e., 20 mg) is effective in avoiding the hyperkalemic effect by pharmacodynamic interaction between esaxerenone and trimethoprim.

This study has many inherent limitations when interpreting the findings. First, its retrospective observational nature leads to bias and confounding factors. Second, it was difficult to assess medication adherence using pill-count due to a retrospective nature although self-reported medication adherence was obtained from individual patients. Third, we could not analyze ΔK according to the esaxerenone dosing schedule as well as co-administration with sodium-glucose co-transporter 2 inhibitors, which is expected to reduce the risk of hyperkalemia through mineralocorticoid receptor antagonists [35]. Fourth, the dataset did not include important information such as diet composition, neurohormonal findings (e.g., renin and aldosterone), and urinary potassium. Finally, most of the patients had chronic kidney disease, which limits the generalizability of the findings.

## Conclusions

The pilot data indicated that the hyperkalemic effect caused by the pharmacodynamic drug–drug interaction between esaxerenone and trimethoprim is weak although we cannot draw a clear conclusion because of a limited sample size. This hyperkalemic effect may increase in patients with reduced renal function or in those administered high doses of trimethoprim. Further study was required to validate the present findings using a large dataset.

### Supplementary Information

Supplementary Material 1.

## Data Availability

The data that support the findings of this study are available on request from the corresponding author.

## Abbreviations

eGFR: Estimated glomerular filtration rate
ΔK: Change in serum potassium levels
ΔNa: Changes in serum sodium levels
ΔBUN: Change in blood urea nitrogen
ΔCr: Change in serum creatinine levels
ΔeGFR: Change in estimated glomerular filtration rate
IQR: Interquartile range
