# Supplementary material for: Hyperkalemic effect of drug–drug interaction between esaxerenone and trimethoprim in patients with hypertension: a pilot study
Source: J Pharm Health Care Sci. 2024 Aug 2;10:46. doi: 10.1186/s40780-024-00366-6 (PMC11295366; doi:10.1186/s40780-024-00366-6)
Supplement: Supplementary file 1 — Supplementary Material 1. [file 40780_2024_366_MOESM1_ESM.docx]

**Additional file 1. Clinical characteristics before propensity score-matching**

| **Variables** | **ESA alone, n=105** | **ESA+TMP, n=8** | **P value** |
| --- | --- | --- | --- |
| **Demographical data** |  |  |  |
| Male, n (%) | 56 (53) | 3 (38) | 0.39 |
| Age, years | 78 [69 to 86] | 72 [64 to 75] | 0.20 |
| Body weight, kg | 64 [55 to 76] | 56 [51 to 78] | 0.24 |
| Body mass index, kg/m^2^ | 25 [23 to 28] | 23 [20 to 26] | 0.14 |
| **Clinical laboratory data** |  |  |  |
| Serum potassium, meq/L | 4.2 [4.0 to 4.4] | 4.2 [4.0 to 5.1] | 0.96 |
| Serum sodium, meq/L | 141 [139 to 142] | 141 [139 to 143] | 0.64 |
| Serum chloride, meq/L | 105 [103 to 106] | 109 [104 to 110] | 0.05 |
| Serum albumin, g/dL | 4.0 [3.6 to 4.3] | 3.4 [2.9 to 3.7] | <0.01 |
| Blood urea nitrogen, mg/dL | 16.4 [13.1 to 22.2] | 21.2 [17.4 to 26.6] | 0.03 |
| Serum creatinine, mg/dL | 0.85 [0.71 to 1.19] | 1.00 [0.74 to 1.31] | 0.55 |
| eGFR, mL/min/1.73m^2^ | 60.5 [42.5 to 76.8] | 50.9 [38.8 to 63.3] | 0.23 |
| **ESA** |  |  | <0.01 |
| 0.625 mg ×1, n (%) | 0 (0) | 1 (13) |  |
| 1.25 mg ×1, n (%) | 52 (50) | 5 (63) |  |
| 2.5 mg ×1, n (%) | 51 (49) | 2 (25) |  |
| 3.75 mg ×1, n (%) | 1 (1) | 0 (0) |  |
| 5.0 mg ×1, n (%) | 1 (1) | 0 (0) |  |
| **TMP** |  |  | - |
| Daily administration, n (%) | - | 1 (13) |  |
| Non-daily administration, n (%) | - | 7 (88) |  |
| **Medications** |  |  |  |
| ARNI or RASi, n (%) | 63 (60) | 6 (75) | 0.40 |
| Loop diuretics, n (%) | 16 (15) | 2 (25) | 0.47 |
| Thiazide diuretics, n (%) | 11 (10) | 1 (13) | 0.86 |
| SGLT2i, n (%) | 18 (17) | 0 (0) | 0.20 |

Abbreviations: ESA, esaxerenone; TMP, trimethoprim; eGFR, estimated glomerular filtration rate; ARNI, angiotensin receptor neprilysin inhibitor; RASi, renin-angiotensin system inhibitor; SGLT2i, sodium-glucose co-transporter 2 inhibitor.

Continuous data are presented as median [interquartile range] and compared using the Mann–Whitney U test. Categorical data are presented as numbers (%) and evaluated using the chi-square test.

$\mathrm{eGFR}\left( \mathrm{mL}/{\min/{{1.73m}^{2}}} \right)=194\times\mathrm{age}^{-0.287}\times{serum creatinine}^{-1.094}\times0.739 \left( if female \right)$ [10]
